# A massive natural disaster, the Great East Japan Earthquake, and the incidence of dialysis due to end-stage kidney disease

**DOI:** 10.1007/s40620-021-01140-9

**Published:** 2021-10-12

**Authors:** Michiaki Abe, Tetsuya Akaishi, Koto Ishizawa, Hirohisa Shinano, Hiroshi Ohtomo, Kazuhiko Orikasa, Shin Takayama, Atsuko Masaura, Mariko Miyazaki, Takaaki Abe, Kenichi Yokota, Tadashi Ishii

**Affiliations:** 1 Department of Education and Support for Regional Medicine, Tohoku University Hospital, Sendai, Japan; 2 Tohoku Medical Megabank Organization, Tohoku University, Sendai, Japan; 3 Division of Nephrology, Endocrinology and Vascular Medicine, Tohoku University Graduate School of Medicine, Sendai, Japan; 4 Kesennuma City Hospital, Kesennuma, Japan

**Keywords:** Massive natural disaster, Dialysis initiation, End-stage kidney disease, Hypertensive renal disease, Diabetic nephropathy

## Abstract

**Background:**

Disaster-related stress can increase blood pressure and the incidence of cardiovascular diseases. However, the role of massive disasters in the development of end-stage kidney disease (ESKD) remains unknown. We investigated the incidence and different causes of dialysis initiation in patients with chronic kidney disease in a city affected by the Great East Japan Earthquake.

**Methods:**

This was a single-center, retrospective observational study. All patients who initiated or were treated with dialysis at Kesennuma City Hospital between 2007 and 2020 were enrolled. The year of dialysis initiation was retrospectively determined based on the initiation date. The causative renal diseases that led to the need for dialysis initiation were divided into four groups: diabetic nephropathy, hypertensive renal disease, glomerulonephritis, and others.

**Results:**

Age at dialysis initiation differed significantly among the four groups (*p* = 0.0262). There was a significant difference in the numbers of the four groups before and after the Great East Japan Earthquake (*p* = 0.0193). The age of hypertensive renal disease patients was significantly higher than those of patients with diabetic nephropathy (*p* = 0.0070) and glomerulonephritis (*p* = 0.0386) after the disaster. The increasing number of dialysis initiations after the Great East Japan Earthquake appeared to be associated with changes in hypertensive renal diseases; the number peaked after 10 years.

**Conclusions:**

There was an increase in the number of dialysis initiations, especially caused by hypertensive renal diseases, for up to 10 years after the Great East Japan Earthquake.

**Graphic abstract:**

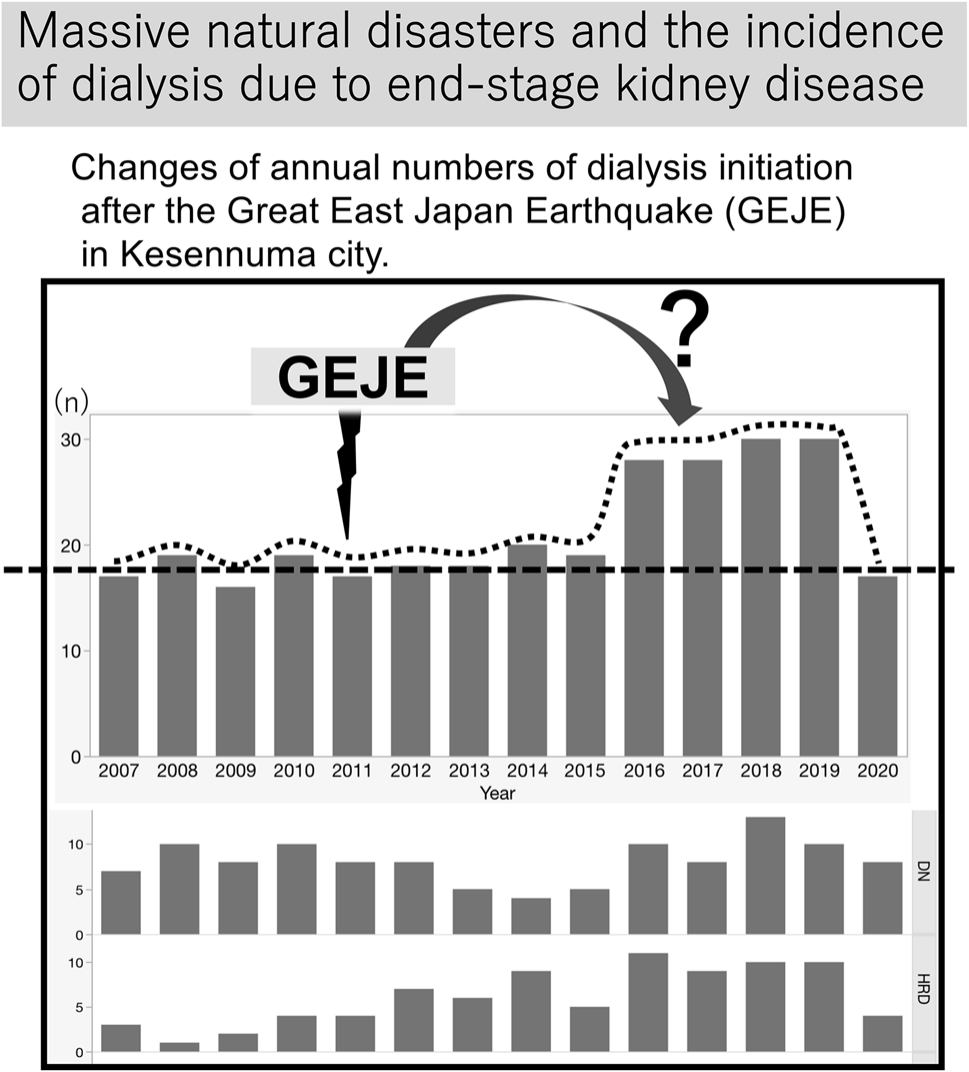

**Supplementary Information:**

The online version contains supplementary material available at 10.1007/s40620-021-01140-9.

## Introduction

Hypertension is a risk factor for chronic kidney disease; furthermore, renal dysfunction also leads to high blood pressure.

Extremely stressful experiences, such as those caused by natural disasters, are commonly known as allostatic loads. The psychological load may disrupt blood pressure and glycemic control [1, 2]. Consequently, poor blood pressure and glycemic control have been reported to increase the risk of cardiovascular events (e.g., heart failure, ischemic heart disease) and stroke in the short term [2–4]. However, the long-term effects of a massive natural disaster are less known and their effects on the development and prognosis of renal diseases have not been elucidated. This study focused on the increase in dialysis initiation after a massive natural disaster, the Great East Japan Earthquake, to evaluate the effects of such disasters on the risk of end-stage kidney disease (ESKD).

In March 2011, Japan was hit by the Great East Japan Earthquake.

This earthquake scored 9.0 on the Richter scale, and was followed by a devastating tsunami along the coastal areas. The disaster caused more than 15,000 casualties, while more than 2500 people have been reported missing as of May 2020. Many cities and towns in the coastal areas, including Kesennuma City (Miyagi, Japan), were almost completely destroyed by the tsunami. Following the Great East Japan Earthquake, Kesennuma City was isolated; Kesennuma City Hospital is the only medical facility in the city with nephrology and dialysis facilities. It is noteworthy that despite a decrease in the population, there was an increase in dialysis initiation several years after the Great East Japan Earthquake [5]. To evaluate the causes of this observation, we studied the causative renal diseases that necessitated dialysis initiation before and after the Great East Japan Earthquake in our remote coastal city.

## Materials and methods

### Patient enrollment

Patients on dialysis initiation in Kesennuma City Hospital were enrolled, as were patients for whom dialysis was initiated in other facilities and returned to Kesennuma for maintenance dialysis.

Exclusion criteria were: dialysis initiation before 2006 and the inability to shift to chronic outpatient dialysis.

All enrolled patients living in Kesennuma City or neighboring towns were victims of the Great East Japan Earthquake, and started dialysis between January 2007 and December 2020. According to the guidelines of the Japanese Society of Dialysis, the following indications to initiate dialysis are retained: serum Creatinine ≥ 8 mg/dL, creatinine clearance (Ccr) ≤ 10 mL/min, difficulty in daily life activities, uncontrollable uremic symptoms. The study was approved by the Ethics Committee of Kesennuma City Hospital (IRB No. 1067). 

### Definition of renal disease groups

The renal diseases that led to the need for dialysis initiation were classified into one of the following four groups: diabetic nephropathy (DN); glomerulonephritis (GN); hypertensive renal disease (HRD), including arteriolosclerosis and arteriosclerotic renal ischemia; and others. Diabetes mellitus, maintained kidney size and high urinary albumin excretion, or renal biopsies were used for diagnosis of DN [6].

Patients with HRD had symptoms of chronic hypertension and low-grade proteinuria (< 1.0 g/gCr) during the mild and moderate CKD phases, usually with atrophic kidneys and without pathological urinary casts. The “other” group included: autosomal dominant polycystic kidney disease, interstitial nephritis, and other unknown causes.

### Statistical analysis

Comparisons of ratios between two or more groups were performed using Fisher’s exact test and the Wilcoxon test. Statistical significance was set at a *p* value of < 0.05. Statistical analyses were performed using JMP^®^ Pro 15.1.0 (SAS Campus Drive, NC, USA).

## Results

Background data of the enrolled patients with dialysis initiation between 2007 and 2020 are summarized in Table 1. The total number of dialysis initiations was 296 (females, 91; age [mean ± SD], 69.1 ± 12.4 years). Two patients underwent preemptive renal transplantation.

**Table 1:** Causal renal diseases for dialysis initiation

	Total	DN	HRD	GN	Others	*p* value
N	296	114	85	54	43	
Gender [female, n]	91	33	31	16	11	0.5816*
Age [years]						
Median	71	70	76	71	71	0.0262**
1st quartile	63	62.75	65	60.75	62	
3rd quartile	78	77	79.5	76.25	81	
Number of patients requiring dialysis initiation before and after the earthquake						
Before GEJE	81	39	13	17	12	0.0193*
After GEJE	215	75	72	37	31	

*GEJE* the Great East Japan Earthquake, *GN* glomerulonephritis, *DN* diabetic nephropathy, *HRD* hypertensive renal disease

*p value by Fisher’s exact test

**p value by Wilcoxon test

Renal diseases were: diabetic nephropathy (n = 114), hypertensive kidney diseases (n = 85), glomerulonephtits (n = 54), and others (n = 43). Fisher’s exact test showed that there was no significant difference in the sex ratio for each causative disease (*p* = 0.5816). The Wilcoxon test showed a significant age difference (*p* = 0.0262). There was a significant difference in the number of cases in each disease group before and after the Great East Japan Earthquake (*p* = 0.0193). In particular, the ratio of hypertensive kidney diseases increased from 16.0% before the disaster to 33.5% thereafter.

The sex and age of the patients according to the causative renal disease groups are shown in Table 2. Laboratory data upon dialysis initiation are reported in Supplementary Table 1.

**Table 2:** Comparisons of gender and age of the causal renal diseases before and after GEJE

	Total	DN	HRD	GN	Others	*p* value
Gender [female; n]						
Before GEJE	26	15	2	7	2	0.2645*
After GEJE	65	18	29	9	9	0.1531*
Age [years]						
Before GEJE						
Median	70	70**	68**	70**	76	0.7430**
1st quartile	59.5	60**	57**	58**	62	
3rd quartile	77	76**	77.5**	76.5**	78	
After GEJE						
Median	72	71**	76**	71**	69	0.0480**
1st quartile	64	64**	68**	61.5**	62	
3rd quartile	78	77**	80**	76.5**	82	

*GEJE* the Great East Japan Earthquake, *GN* glomerulonephritis, *DN* diabetic nephropathy, *HRD* hypertensive renal disease

*p value by Fisher’s exact test

**p value by Wilcoxon test; DN vs. HRD, *p* = 0.0070; HRD vs. GN, *p* = 0.0386

**Fig. 1:**
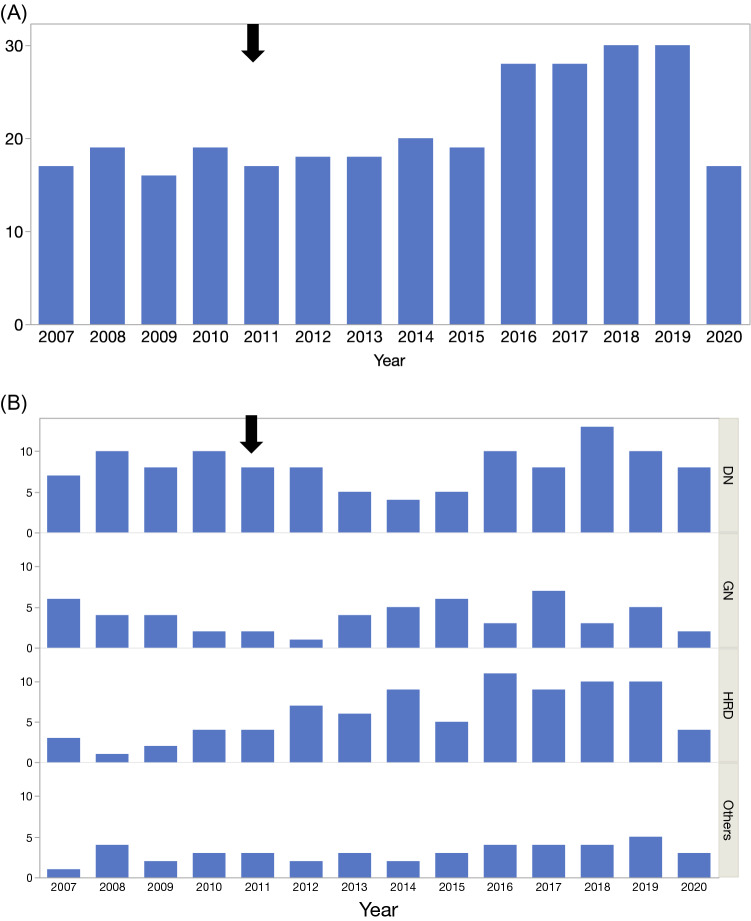
**A** Changes of annual numbers of dialysis initiation in Kesennuma city. **B** Changes of annual numbers of dialysis initiation based on the four causal renal diseases. A thick arrow indicates the occurrence of GEJE. *X*-axis is year and *Y*-axis is number. *DN* diabetic nephropathy, *HRD* hypertensive renal disease, *GN* glomerulonephritis

Figure 1A shows the number of dialysis initiations before and after the Great East Japan Earthquake. The numberage atof dialysis initiation by four causal renal diseases is shown in Fig. 1B. Notably, the increase in dialysis initiation due to hypertension reached a plateau 5 years after the Great East Japan Earthquake, and returned to the before level 10 years later.

## Discussion

This study shows a significant increase in the number of dialysis initiations 5 years after the Great East Japan Earthquake, which was sustained for a decade.

This increase was particularly associated with hypertensive renal diseases. Hypertension is a major, treatable cause of chronic kidney disease [7, 8]. As the African American Study of Kidney Disease and Hypertension Collaborative Research Group has reported, renal function continues to deteriorate even with well-controlled blood pressure and with low proteinuria in hypertensive patients [9]. Stress associated with disasters, especially if associated with a sense of loss, low income, and malnutrition, causes sustained activation of the sympathetic nervous system, which is a risk factor for the development and (poor) management of hypertension. Sodium intake may likewise be increased. This type of hypertension is known as disaster hypertension [10–12]. The increased frequency of cardiovascular events immediately after a natural disaster is attributed to the hyperactivation of the sympathetic nervous system, which increases blood pressure, and induces both endothelial dysfunction and hyperglycemia [1, 2]. Inadequate blood pressure control is detrimental to the vascular system, enhancing the deterioration of renal function and arteriosclerosis [13, 14]. In line with this, hypertensive renal diseases needing dialysis increased after the Great East Japan Earthquake, as shown for the first time in our results [15]. While diabetic kidney disease remains a major cause of dialysis initiation, it did not share this increase.

A further reason for the increased incidence of hypertensive renal diseases after the Great East Japan Earthquake in Kesennuma City may be the aging of its citizens, while the younger population decreased, in particular after the earthquake. Overall, need for dialysis start increased (*p* = 0.0317) mainly due to hypertensive disorders (odds ratio post vs pre-earthquake: 2.52, *p* = 0.0037) (Supplementary Table 2), and the increase was higher in the elderly (Supplementary Figures A, B, E, F).

This study has several limitations. First, data concerning the patients’ blood pressure levels in the initial period after the Great East Japan Earthquake were not available. Nonetheless, we must consider that poor blood pressure was common in our in area for at least 1 year after the earthquake, which is in line with logistical constraints and psychological distress, both of which increase the risk of non-compliance [16]. Second, the role of the aging process is not clear. Third, this study was based on data from a single hospital. This is however the first study to suggest that a large natural disaster could lead to an increase in end stage kidney disease, and in particular due to hypertensive nephropathies.

This study also highlights the importance of conducting further studies on this topic, focusing on the long-term assessment of renal function and blood pressure after massive natural disasters.

## Supplementary Information

Below is the link to the electronic supplementary material.

Supplementary file1 (XLSX 22 kb)

Supplementary file2 (DOCX 18 kb)

Supplementary file3 (DOCX 19 kb)

Supplementary file4 (DOCX 247 kb)
