# Supplementary material for: A massive natural disaster, the Great East Japan Earthquake, and the incidence of dialysis due to end-stage kidney disease
Source: J Nephrol. 2021 Oct 12;35(3):719–24. doi: 10.1007/s40620-021-01140-9 (PMC8995295; doi:10.1007/s40620-021-01140-9)
Supplement: Supplementary file 2 — Supplementary file2 (DOCX 18 kb) [file 40620_2021_1140_MOESM2_ESM.docx]

**Supplementary Table 1.** Laboratory date at their dialysis initiation from Jan. 2017 to Dec. 2020.

Blood examination

|  | n | Cr,  mg/dL | BUN,  mg/dL | Hb,  mg/dL | SBP,  mmHg | DBP,  mmHg | HR,  bpm |
| --- | --- | --- | --- | --- | --- | --- | --- |
| DN | 20 | 8.47 +0.51 | 95.9 +6.3 | 9.74 +0.37 | 144 +4.3 | 71 +2.9 | 75+2.9 |
| HRD | 23 | 8.44 +0.47 | 109.3 +5.9 | 10.47 +0.35 | 153 +4.0 | 69 +2.6 | 78 +2.8 |
| GN | 6 | 8.50 +0.93 | 122.3 +11.6 | 9.50 +0.65 | 140 +8.0 | 75 +5.3 | 76 +5.4 |
| Other | 9 | 10.52 +0.76 | 97.6 +9.5 | 9.61 +0.57 | 144 +6.3 | 82 +4.2 | 77 +4.3 |

[mean + SD]

Cr; creatinine, BUN; blood urea nitrogen, Hb; hemoglobin, SBP; systolic blood pressure, DBP; diastolic blood pressure, HR; heart rate.

Urinalysis

|  | n | Proteinuria | | | | Urine occult blood | | | |
| --- | --- | --- | --- | --- | --- | --- | --- | --- | --- |
|  |  | + | 2+ | 3+ | 4+ | - | +/- | 2+ | 3+ |
| DN | 19 | 1 | 5 | 11 | 2 | 3 | 5 | 7 | 4 |
| HRD | 21 | 2 | 10 | 8 | 1 | 1 | 0 | 3 | 1 |
| GN | 5 | 1 | 1 | 3 | 0 | 1 | 0 | 3 | 1 |
| Other | 8 | 1 | 2 | 4 | 1 | 1 | 3 | 4 | 0 |

The blood examination, urinalysis and blood pressure at the time when the patient decided to initiate dialysis in Kesennuma City Hospital from 2017 to 2020 (Supplementary data). About a half of dialysis initiations were decided in Kesennuma City Hospital. Many patients of GN containing RPGN or sudden onset of decompensated heart failure with CKD by any causes were introduced to neighbor big advanced treated hospitals and came back after treatment of the dialysis initiation in case of advanced-worsen renal function. During several months follow-up on Kesennnuma City hospital for measurement of the timing of dialysis initiation after the surgery of arterial-venous fistula for preparation of hemodialysis, qualitative urinalysis was performed but quantitative urinary proteinuria excretion was not performed except for GN and nephrosis. Proteinuria happened to be predominant in all patients with end-stage kidney diseases already due to massively destructed glomerular tufts and uncontrolled high blood pressure. Some patients of emergent dialysis initiations due to sudden onset of congestive heart failure and so on were not performed urinalysis at the time.
