# Supplementary material for: A massive natural disaster, the Great East Japan Earthquake, and the incidence of dialysis due to end-stage kidney disease
Source: J Nephrol. 2021 Oct 12;35(3):719–24. doi: 10.1007/s40620-021-01140-9 (PMC8995295; doi:10.1007/s40620-021-01140-9)
Supplement: Supplementary file 3 — Supplementary file3 (DOCX 19 kb) [file 40620_2021_1140_MOESM3_ESM.docx]

**Supplementary Table 2.** Logistic regression tests for effects of age, gender, and GEJE on the causal renal diseases and each of the diseases. Abbreviations; GEJE, the Great East Japan Earthquake; HRD, hypertensive renal disease; DN, diabetic nephropathy; GN, glomerulonephritis.

| Causal renal diseases | Likelihood ratio chi-square | *p* |
| --- | --- | --- |
| GEJE | 8.83 | 0.0317* |
| Age | 6.91 | 0.0748 |
| Gender | 2.32 | 0.5089 |

Whole model test; *p*=0.0238*, Lack of fit; *p*=0.4743

**p*<0.05

| HRD | Odds ratio (95% CI) | *p* |
| --- | --- | --- |
| GEJE (after) | 2.523 (1.3027–4.8875) | 0.0037** |
| Age (+1) | 0.981 (0.9596–1.0028) | 0.0805 |
| Gender (f) | 0.928 (0.5299–1.6265) | 0.7947 |

Whole model test; *p*=0.0057**, Lack of fit; *p*=0.5867

***p*<0.01

| DN | Odds ratio (95% CI) | *p* |
| --- | --- | --- |
| GEJE (after) | 0.5965 (0.3540–1.0051) | 0.0528 |
| Age (+1) | 1.0138 (0.9947–1.0333) | 0.1590 |
| Gender (f) | 1.0594 (0.6353–1.7667) | 0.8251 |

Whole model test; *p*=0.0962, Lack of fit; *p*=0.0743

| GN | Odds ratio (95% CI) | *p* |
| --- | --- | --- |
| GEJE (after) | 0.781 (0.4097–1.4898) | 0.4578 |
| Age (+1) | 0.998 (0.9739–1.0224) | 0.8601 |
| Gender (f) | 1.277 (0.6843–2.3814) | 0.4463 |

Whole model test; *p*=0.7620, Lack of fit; *p*=0.5604
