# Supplementary material for: A massive natural disaster, the Great East Japan Earthquake, and the incidence of dialysis due to end-stage kidney disease
Source: J Nephrol. 2021 Oct 12;35(3):719–24. doi: 10.1007/s40620-021-01140-9 (PMC8995295; doi:10.1007/s40620-021-01140-9)
Supplement: Supplementary file 4 — Supplementary file4 (DOCX 247 kb) [file 40620_2021_1140_MOESM4_ESM.docx]

**Supplementary Figure.** Relationships of annual dialysis initiation ratio (per 10,000) with causal renal diseases before and after the GEJE.

| 1. Annual ratio before GEJE.     *p*=0.0194† | 1. Annual ratio after GEJE.     *p*=0.0005†† |
| --- | --- |
| 1. Among the middle aged (≥15, <65) before GEJE.   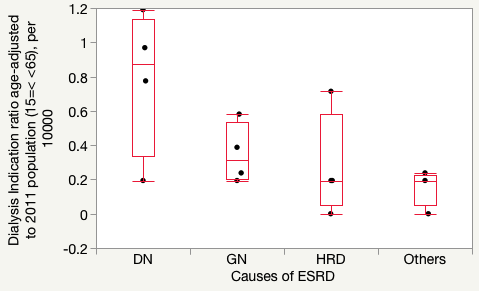  *p*=0.1174 | 1. Among the middle aged (≥15, <65) after GEJE.     *p*=0.0094†† |
| 1. Among the elderly (≥65) before GEJE.     *p*=0.0492† | 1. Among the elderly (≥65) after GEJE.   *p*=0.0007†† |

**Legend.**

**Supplementary Figure.** Relationships of annual dialysis initiation ratio (per 10,000) with causal renal diseases before and after GEJE.

Relationships of annual dialysis initiation ratios, per 10,000, among causal renal diseases were calculated. The population of Kesennuma City was referred to the open-public report of the Census (https://www.kesennuma.miyagi.jp/sec/s021/010/020/H28-9/20161014163117.html]. The annual dialysis indication ratios were adjusted to the 2011-year population and are shown before (A) and after the GEJE (B). Furthermore, the annual dialysis indication ratio was adjusted to the 2011-year three-division population: younger (<15), middle (≥15 <65, C, D), and elderly (≥65, E, F) before (C, E) or after the GEJE (D, F). There was no indication for dialysis in children under 15 years of age. The Japanese census was conducted every five years. However, the population adjustment in 2020 was based on data from 2015 for this analysis, because the latest three-division population has not yet been reported. The statistical analysis was performed using the Wilcoxon test (**p*<0.05, ***p*<0.01) and Kruskal-Wallis test (†*p*<0.05, ††*p*<0.01). Abbreviations: GEJE, Great East Japan Earthquake; HRD, hypertensive renal disease; DN, diabetic nephropathy; GN, glomerulonephritis.
